# MET-Driven Resistance to Sotorasib in KRAS G12C–Mutant NSCLC and Response to Combined KRAS and MET Inhibition

**DOI:** 10.1016/j.jtocrr.2025.100925

**Published:** 2025-10-27

**Authors:** Richard Riedel, Lea Ruge, Malte Verheyen, Felix John, Heather Scharpenseel, Lucia Nogova, Sebastian Michels, Rieke N. Fischer, Anna Eisert, Carolin Jakob, Emanuel Niesen, Jana Fassunke, Janna Siemanowski-Hrach, Carina Heydt, Anne Bunck, Udo Siebolts, Sabine Merkelbach-Bruse, Reinhard Buettner, Jürgen Wolf, Matthias Scheffler

**Affiliations:** a Department I of Internal Medicine, University Hospital Cologne, Cologne, Germany; b Center for Integrated Oncology, University Hospital Cologne, Cologne, Germany; c Faculty of Medicine, University of Cologne, Cologne, Germany; d Lung Cancer Group Cologne, Cologne, Germany; e Department of Pathology, Molecular Pathology, University Hospital Cologne, Cologne, Germany; f Department of Radiology, University Hospital Cologne, Cologne, Germany

**Keywords:** NSCLC, *KRAS G12C*, Sotorasib, *MET* amplification, Tyrosine kinase inhibitor resistance

## Abstract

**Introduction:**

*KRAS G12C* mutations define a molecularly distinct subset of NSCLC for which targeted therapy with sotorasib has exhibited clinical efficacy. However, acquired resistance is inevitable. *MET* amplification has been described as a putative off-target resistance mechanism, although its clinical relevance remains incompletely understood.

**Methods:**

We conducted a retrospective case series of patients with *KRAS G12C*–mutant NSCLC treated with sotorasib at the University Hospital Cologne, Germany. Patients with available paired pre- and posttreatment biopsies were analyzed for resistance mechanisms using routine molecular diagnostics, including *MET* fluorescence in situ hybridization.

**Results:**

Nine patients with paired pre and posttreatment biopsies were identified. High-level *MET* amplification was detected by fluorescence in situ hybridization in four cases and intermediate-level amplification in one case after progression on sotorasib. Notably, one patient with acquired *MET* amplification achieved a renewed partial response to the combination of sotorasib and tepotinib after progression on sotorasib monotherapy.

**Conclusion:**

This study provides real-world evidence that *MET* amplification is an acquired and potentially targetable resistance mechanism to KRAS G12C inhibition in NSCLC. Our findings support rebiopsy at progression on sotorasib. Further prospective trials are warranted to validate *MET* amplification as a resistance mechanism and to define optimal therapeutic thresholds for combined KRAS and MET inhibition.

## Introduction

Advances in molecular profiling in NSCLC have enabled the identification of actionable oncogenic drivers, fundamentally reshaping treatment paradigms in advanced disease. Among these drivers, *KRAS* G12C mutations—found in approximately 13% to 5% of lung adenocarcinomas—represent a distinct molecular subset^1^^,^^2^ that has recently been added to the family of targetable aberrations. However, despite these therapeutic advances, acquired resistance is the main challenge with targeted therapy in NSCLC.

Sotorasib, a first-in-class, covalent KRAS G12C inhibitor that binds to the guanosine diphosphate–bound inactive form of KRAS, has exhibited clinical activity in patients with advanced *KRAS G12C*-mutant NSCLC. Sotorasib—and the KRAS G12C inhibitor adagrasib—have been found to prolong progression-free survival than docetaxel in pretreated patients, and have gained regulatory approvals in multiple countries.^3^^,^^4^

Recently, the armamentarium of drugs targeting KRAS has been expanded with the introduction of inhibitors targeting the GTP-bound form of KRAS.

As expected, acquired resistance to KRAS G12C inhibition inevitably develops. Resistance mechanisms are heterogeneous and include both on-target mutations (e.g., secondary *KRAS* alterations)^5^^,^^6^ and off-target pathways.^7^ Among these, *MET* amplification has emerged as an off-target resistance mechanism, capable of by passing *KRAS-*dependent signaling.^8^^,^^9^ Although observed in preclinical models and isolated case reports, the real-world incidence and clinical impact of *MET* amplification as a resistance mechanism to sotorasib treatment remain incompletely characterized, and there is limited clinical evidence to guide treatment recommendations for acquired *MET* amplification.

In this retrospective case series, we examined patients with advanced *KRAS G12C*–mutant NSCLC who developed disease progression after treatment with sotorasib. We explore the emergence of *MET* amplification as a potential mechanism of acquired resistance and explore the therapeutic potential of combined KRAS and MET inhibition to overcome *MET*-mediated resistance.

## Materials and Methods

We retrospectively identified patients with NSCLC KRAS *G12C* mutation and sotorasib treatment in any line of therapy at the University Hospital Cologne between January 2015 and December 2024. We identified those with paired pre- and postsotorasib biopsies.

All patients gave informed consent according to Good Clinical Practice and local standards.

Tumor samples were analyzed as part of routine diagnostics on the platform of the National Network Genomic Medicine.

Detailed descriptions of sequencing methods and fluorescence in situ hybridization (FISH) are provided in the Supplementary Appendix.

## Results

### Patient Characteristics

We identified nine patients (January 2015 to December 2024) with NSCLC, KRAS *G12C* as the primary driver, treatment with sotorasib in any line of therapy, and paired pre- and posttreatment biopsies. Six patients were male, and three patients were female. The median age at diagnosis was 63 years (range 48–68). Eight patients had adenocarcinoma, and one patient (#6) had large-cell neuroendocrine carcinoma. Four of the patients were former or current smokers; the smoking status of the remaining five patients was unknown. Seven patients presented with stage IV at initial diagnosis. (Table 1).

**Table 1:** Initial Molecular Characteristics and Treatment Outcomes of Patients With Paired Pre- and Post-Treatment Biopsies

Patient number	Sex	Age at initial diagnosis (y)	Smoking status	Stage at initial diagnosis (UICC 8th edition)	Histological diagnosis	MET amp	PD-L1 (TPS in %)	Co-occuring mutations	Sotorasib line of therapy	Best response to sotorasib	DOT with sotorasib (mo)	OS (mo)
#1	M	48	Current	IVA	Adeno	None	5	TP-53	3	PR	Ongoing	Alive
#2	M	56	Former	IVB	Adeno	None	40	None	4	SD	5	36
#3	M	63	Unknown	IIB	Adeno	None	0	CTNNB1	2	PR	13.9	Alive
#4	M	67	Former	IVA	Adeno	N/A	8	None	2	PR	15	33
#5	F	68	Unknown	IVA	Adeno	None	0	KEAP1 STK11	2	SD	7.6	LTFU
#6	M	61	Unknown	IVA	LCNEC	None	3	None	3	PD	2	62
#7	F	65	Unknown	IVB	Adeno	N/A	0	None	2	PD	4	20.7
#8	F	56	Unknown	IVA	Adeno	N/A	1	None	2	PR	30.5	46.5
#9	M	68	Current	IIIA	Adeno	None	100	None	3	PR	29.5	56.4

Adeno, adenocarcinoma; amp, amplification; DOT, duration of therapy; F, female; LCNEC, large cell neuroendocrine carcinoma; LTFU, lost to follow-up; M, male; MET, hepatocyte growth factor recepto; N/A, not applicable; OS, overall survival from diagnosis of stage IV NSCLC; PD, progressive disease; PR, partial response; SD, stable disease; TPS, tumor proportion score; UICC, Union internationale contre le cancer.

### Initial Molecular Findings, Programmed Death-Ligand 1 Status, and Course of Treatment

Two patients had tumors negative for programmed death-ligand 1 (PD-L1), five patients had a PD-L1 expression (tumor proportion score) between 1% and 50% and one patient (#9) had a PD-L1 expression of greater than 50%. Six patients had no co-occurring mutations aside from *KRAS G12C*. One patient had a co-occurring *CTNNB1* mutation, one patient had a *TP53* mutation, and one patient had *KEAP1*- and *STK11* mutations. *MET* FISH was performed at initial diagnosis in six cases. None of these patients exhibited a *MET* amplification. A summary of the results of molecular analyses from all nine patients is provided in Supplementary Table 1.

First-line treatment for stage IV NSCLC consisted of chemoimmunotherapy (platinum-based chemotherapy plus immune-checkpoint blockade) in eight patients. One patient received platinum-based chemotherapy alone, as treatment was started before immune-checkpoint blockade was approved for first-line use in Germany; this patient later received nivolumab monotherapy second-line. Three patients were treated with second-line monochemotherapy (with or without VEGF blockade). Most patients (five) received sotorasib as second-line treatment; three patients got sotorasib as third-line treatment, and one patient as fourth-line treatment.

Sotorasib led to progressive disease (PD) in two cases, stable disease in two cases, and partial response (PR) in five cases. In patients with stable disease or PR, the duration of therapy ranged from 5 to 30 months and was ongoing (beyond progression) in one case at the time of analysis (Table 1).

### Postsotorasib Biopsies

*MET* FISH was performed in all nine postsotorasib tumor biopsies. High-level *MET* amplification was detected in four cases. Three of these amplifications were acquired; in one case (#7), *MET* FISH could not be performed from the initial tumor biopsy. One patient had an acquired intermediate-level *MET* amplification, and three patients had no *MET* amplification (Table 2). One patient had a novel *CTNNB1* mutation, one patient had a novel *IDH2* mutation, and two patients had novel *TP53* mutations.

**Table 2:** FISH Results From Patients With Paired Pre- and Post-Treatment Biopsies and Side of Primary Biopsy and Rebiopsy

Patients	Pat. #1		Pat. #2		Pat. #3		Pat. #5		Pat. #6		Pat. #9	
Biopsy site	Primary lung tumor		Primary lung tumor		Pleural nodule		Primary lung tumor		Primary lung tumor		Primary lung tumor	
Pre-treatment *MET* FISH	GCN	MET/CEN7	GCN	MET/CEN7	GCN	MET/CEN7	GCN	MET/CEN7	GCN	MET/CEN7	GCN	MET/CEN7
	2.6	1.14	3.17	1.17	2.5	1.39	1.75	0.83	1.82	0.98	3.18	1.34
	No amp		No amp		No amp		No amp		No amp		No amp	

Rebiopsy site	Adrenal gland		Pleural effusion		Mediastinal lymph node		Primary lung tumor		Liver metastasis		Pleural nodule	
Post-treatment *MET* FISH	GCN	MET/CEN7	GCN	MET/CEN7	GCN	MET/CEN7	GCN	MET/CEN7	GCN	MET/CEN7	GCN	MET/CEN7
	8.1	0.89	5.95	2.2	5.13	1.23	2.3	1.35	2.35	1.07	9.6	2.09
	High-level amp		High-level amp		Intermediate-level amp		No amp		No amp		High-level amp	

Amp, amplification; FISH, fluorescent in situ hybridization; MET/CEN7, MET to centromer 7 ratio; *MET* FISH. GCN, MET gene copy number; pat, patient.

### Response to Combined KRAS and MET Inhibition

Patient #9 was a 68-year-old male patient initially diagnosed with right upper lobe adenocarcinoma in 2017. After bilobectomy and adjuvant chemoradiotherapy, the patient had recurrent disease in the right hilar region with pulmonary and pleural metastases.

He was treated with carboplatin, pemetrexed, and pembrolizumab, resulting in a PR. After PD on maintenance therapy with pembrolizumab and pemetrexed, he received docetaxel and ramucirumab for approximately 7 months, with stable disease as the best response. When the bipulmonary metastases exhibited further progression, therapy was switched to sotorasib as the molecular analysis of the initially resected tumor exhibited *KRAS G12C*. *MET* FISH revealed no *MET* amplification (*MET/CEN7* 1.34; GCN 3.18). *TP53* was presented as wild type.

**Figure 1:**
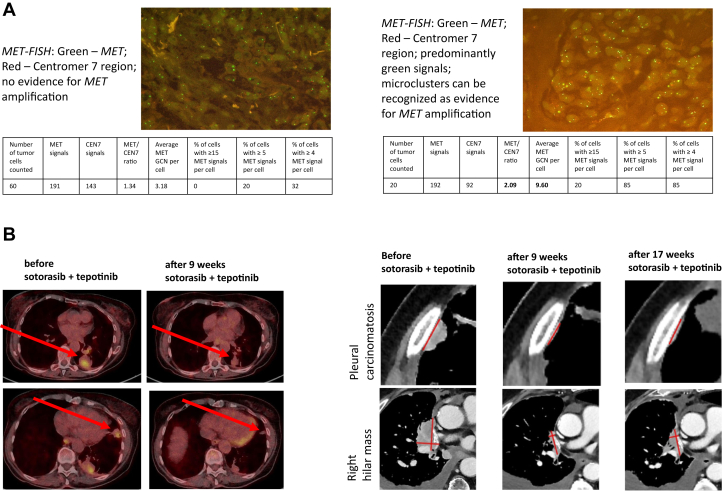
Patient #9. A: representative FISH images before and after treatment with sotorasib B: FDG-PET-and CT-images of patient #9 before and after start of treatment with sotorasib + tepotinib showing a confirmed partial response (PR) according to RECIST 1.1. CT, computed tomography; FDG-PET, fluorodeoxyglucose positron emission tomography; FISH, fluorescent in situ hybridization; RECIST, response evaluation criteria in solid tumors.

Sotorasib led to a PR. Because of intermittent diarrhea, the sotorasib dose was reduced to 600 mg/day. After approximately 2 years, the patient again exhibited PD of bipulmonary metastases, new and growing pleural lesions, and a progressive right hilar tumor mass. Rebiopsy of a growing pleural lesion again revealed the known *KRAS G12C* and a *TP53* mutation. *MET* FISH now revealed an acquired high-level *MET* amplification (*MET/CEN7* 2.09; GCN 9.6). A combined therapy with sotorasib 600 mg/day and tepotinib 450 mg/day was started. This led to a confirmed PR with the best response of pleural lesions and the right hilar tumor mass (Fig. 1). The combination of sotorasib and tepotinib was overall well-tolerated. Laboratory analyses revealed no evidence of high-grade toxicities, especially no hepatotoxicity was observed. Clinically, the patient developed peripheral edema of the forearms, hands, and ankles (Common Terminology Criteria for Adverse Events grade 2), which led to a reduction of the tepotinib dose to 225 mg once daily after 4 months at patient’s request. After about 7 months of therapy, the patient again exhibited pulmonary and pleural PD and died of community-acquired pneumonia.

## Discussion

This retrospective case series provides real-world evidence supporting *MET* amplification as a clinically relevant mechanism of acquired resistance to sotorasib in NSCLC. Among nine patients with advanced *KRAS* G12C-mutant NSCLC, we identified high-level *MET* amplification in four cases and intermediate-level *MET* amplification in one case after disease progression on sotorasib according to the criteria of Schildhaus et al.^10^ Of these five patients, four did not have *MET* amplification at baseline, highlighting the acquired nature of this resistance mechanism. Of note, one patient (#7) without an initial response to sotorasib had high-level *MET* amplification in the posttreatment biopsy. As no MET FISH analysis could be performed on the baseline specimen, it remains uncertain whether this amplification was acquired. If present at baseline, this finding may indicate that *MET* amplification can also serve as a mechanism of intrinsic resistance to KRAS inhibition, although this cannot be definitively established from our data.

Our findings are consistent with preclinical and clinical data suggesting that *MET* amplification functions as a bypass resistance mechanism. Subsequent translational analyses of patients with *KRAS* G12C–mutant NSCLC treated with adagrasib described multiple mechanisms of resistance, including *MET* amplification, which was identified in two out of 10 patients.^8^ Recently, in a series of 20 patients with PD after combined therapy with carboplatin, pemetrexed, and sotorasib in a prospective phase 2 trial, acquired *MET* amplification was detected in one case by analyzing circulating deoxyribonucleic acid.^11^ The rate of acquired *MET* amplification in our cohort is higher. It should be noted that no FISH analysis was performed in the mentioned trial, which makes comparability difficult. In addition, the data must be interpreted with caution because of the small number of cases and a possible selection bias caused by the retrospective nature of our cohort.

The clinical relevance of *MET* amplification is illustrated by one patient in our cohort who benefited from combination therapy with sotorasib and tepotinib. After initial response and later progression on sotorasib monotherapy, this patient got high-level *MET* amplification and subsequently achieved another PR on initiation of dual KRAS/MET inhibition. This case supports preclinical data indicating potential synergy between KRAS G12C inhibitors and MET inhibitors in *MET*-amplified, sotorasib-resistant tumors^9^ and represents, to the best of our knowledge, the first case of a successful clinical treatment in this setting. These findings may have direct therapeutic implications by suggesting that MET-directed combination strategies may overcome a key resistance mechanism to KRAS G12C inhibition. Prospective data and larger patient numbers are needed to substantiate these findings. In addition, *MET* amplification is a variable biomarker, and further research is needed to determine which cutoffs best predict response to combined KRAS/MET inhibition. Whereas high gene copy numbers (≥10) seem to be necessary for successful treatment of patients with *MET* amplification as a primary driver,^12^ in the treatment of patients with *EGFR*-mutated NSCLC and acquired *MET* amplification as a resistance mechanism to EGFR TKI therapy, *MET* gene copy numbers greater than or equal to 5 may be sufficient for successful combined EGFR and MET inhibition.^13^

Our study has several limitations. The sample size is small, reflecting the rarity of paired biopsies in clinical practice. The retrospective nature of the analysis may introduce selection bias, and treatment decisions were not standardized. Sotorasib was used in different lines of therapy, which leads to additional heterogeneity in the cohort. Furthermore, some potential resistance mechanisms are not represented by the routine analyses we used (e.g., acquired *KRAS* amplification, acquired fusions), and no circulating deoxyribonucleic acid was analyzed, which could have revealed additional resistance mutations. Our cohort also seems to be biased, with five out of nine patients responding to sotorasib, which exceeds the response rates in randomized trials.

## Conclusion

Our results provide additional evidence that *MET* amplification is a relevant and potentially targetable mechanism of acquired resistance to sotorasib in patients with *KRAS G12C*-mutant NSCLC. These findings support rebiopsy at progression and highlight the need for broader molecular testing beyond *KRAS* to inform treatment sequencing. Combined KRAS and MET inhibition may represent a rational next-line strategy in selected patients and should be further explored in prospective trials.

## Disclosure

Richard Riedel received consulting fees from Johnson and Johnson and support for attending meetings from Johnson and Johnson. His institution received research support from Johnson and Johnson, BMS, Novartis, Pfizer, MSD, Amgen and Dracen. Lea Ruge received consulting fees from Amgen and support for attending meetings from Amgen and Johnson and Johnson and participated Advisory Boards from Johnson and Johnson. Her institution received research support from Johnson and Johnson, BMS, Novartis, Pfizer, MSD, Amgen and Dracen. Malte Verheyen, his institution received research support from Johnson and Johnson, BMS, Novartis, Pfizer, MSD, Amgen and Dracen. Felix John received consulting fees from Boehringer Ingelheim, Reesi and Johnson and Johnson. He received research funding from Astra Zeneca and Amgen. His institution received research support from Johnson and Johnson, BMS, Novartis, Pfizer, MSD, Amgen and Dracen. Heather Scharpenseel, received support for attending meetings from Johnson and Johnson. Her institution received research support from Johnson and Johnson, BMS, Novartis, Pfizer, MSD, Amgen and Dracen. Lucia Nogova received honoraria from Pfizer, Roche, Johnson and Johnson and Astra Zeneca and support for attending meetings from Roche, Pfizer and Johnson and Johnson. She participated Advisory Boards from BMS, Roche, Johnson and Johnson, Pfizer and Astra Zeneca. Her institution received research support from Johnson and Johnson, BMS, Novartis, Pfizer, MSD, Amgen and Dracen. Sebastian Michels, his institution received research support from Johnson and Johnson, BMS, Novartis, Pfizer, MSD, Amgen and Dracen. Rieke N. Fischer received Honoraria from Johnson and Johnson. Her institution received research support from Johnson and Johnson, BMS, Novartis, Pfizer, MSD, Amgen and Dracen. Anna Eisert received honoraria from Merck, Amgen, Astra Zeneca and Takeda. Her institution received research support from Johnson and Johnson, BMS, Novartis, Pfizer, MSD, Amgen and Dracen. Carolin Jakob, her institution received research support from Johnson and Johnson, BMS, Novartis, Pfizer, MSD, Amgen and Dracen. Emanuel Niesen has no conflict to declare. Jana Fassunke received honoraria from BMS. Janna Siemanowski-Hrach received honoraria from Biocartis, Targos, Merck, Astra Zeneca und Molecular Health. Carina Heydt received honoraria from Novartis and Molecular Health. Anne Bunck has no conflict to declare. Udo Siebolts has no conflict to declare. Sabine Merkelbach-Bruse received honoraria from Amgen, Astra Zeneca, Roche, Novartis, DLS, QuIP GmbH, BMS, Illumina, Daiichi-Sankyo and Stemline. She received third party funding from Astra Zeneca, Roche, Zytovision and QuIP GmbH. Reinhard Buettner received honoraria from AbbVie, Amgen, Astra Zeneca, Bayer, BMS, Boehringer-Ingelheim, Illumina, Johnson and Johnson, Lilly, Merck-Serono, MSD, Novartis, Qiagen, Pfizer, Roche and Targos MP inc. Jürgen Wolf received honoraria from Astra Zeneca, Bayer, Blueprint, BMS, Boehringer Ingelheim, Chugai Europe, Daiichi-Sankyo, Disco Pharmaceuticals, Ellipses Pharma, Genmab, Merck, Mirati, Johnson and Johnson, Lilly, Loxo, MSD, Novartis, Nuvalent, Pfizer, Pierre-Fabre, Regeneron, Roche, Seattle Genetics, Takeda, Turning Point, Zuelling Pharma, AbbVie, Amgen, Beigene. His institution received research support from Johnson and Johnson, BMS, Novartis, Pfizer, MSD, Amgen and Dracen. Matthias Scheffler received institutional research support from Dracen Pharmaceuticals, speaker grants from Amgen, Astra Zeneca, Boehringer Ingelheim, Johnson and Johnson, Novartis, Pfizer, Roche, Sanofi-Aventis, Siemens Healthineers, Takeda and BMS. He received support for attending meetings from Johnson and Johnson and Pfizer. He joined the Climate Change Task Force of ESMO and the Lung Cancer Group of the EORTC. His institution received research support from Johnson and Johnson, BMS, Novartis, Pfizer, MSD, Amgen and Dracen.
