# Supplementary material for: MET-Driven Resistance to Sotorasib in KRAS G12C–Mutant NSCLC and Response to Combined KRAS and MET Inhibition
Source: JTO Clin Res Rep. 2025 Oct 27;7(3):100925. doi: 10.1016/j.jtocrr.2025.100925 (PMC12971985; doi:10.1016/j.jtocrr.2025.100925)
Supplement: Supplementary Material [file mmc1.docx]

**Supplementary Appendix**

**Collection of data and response evaluation**

All patients consented to the procedures according to Good Clinical Practice and local standards. Clinical features were taken from the patient's medical record and histopathological characteristics from the local pathologist's documentation. Clinical staging was done by the treating physicians according to UICC 8. Edition.

Response to therapy was assessed according to RECIST 1.1. if applicable [1,2].

The duration of therapy (DOT) was defined as the time interval between initiation and the last administration of therapy.

**Next Generation Sequencing (NGS)**

NGS was performed on all formalin-fixed, paraffin-embedded samples with validated gene panels using either AmpliSeq gene panels until 2015 (Thermo Fisher Scientific, Waltham, MA; LUN3, 14 genes) or GeneRead or Twist gene panels, respectively, from 2015 further on (Qiagen, Hilden, Germany; LUN4, 17 genes; LUN5, 19 genes; Twist Biosciences, nNGM 2.0, 26 genes, nNGM 3.2, 30 genes, Supplementary Table S2) as described previously [3].

Genomic variants were called using a bioinformatic in-house algorithm [4].

All parallel sequencing steps were performed on the MiSeq or NextSeq platform from Illumina (Illumina, San Diego, CA, USA).

**Fluorescence in situ hybridization (FISH)**

FISH analysis was performed on 4 µm thick slides prepared from the same FFPE blocks used for NGS. Probes specific to the regions of interest were obtained from ZytoVision. The procedure followed the manufacturer’s instructions, with hybridization conditions optimized for each probe set. Fluorescence signals were detected using a Microscope KP-PLUS slides (Klinipath, Duiven, Netherlands). Tissue slides were hybridized overnight with the Zyto-Light SPEC Dual Color Probe (ZytoVision, Bremerhaven, Germany) — *MET*/CEN7. Twenty contiguous tumor cell nuclei were individually evaluated to calculate the gene/centromere (CEN) ratio and the average gene copy number (GCN) per cell. Signals were manually counted in cells with ≤15 copies and estimated using clusters for 15 and more gene copies.

In cases of low or intermediate results, an additional forty cells from different areas were evaluated.

Patients were classified into the following four groups of MET amplification status [5]:

1. *High-level amplification* was defined in tumors with

a. a MET/CEN7 ratio ≥2.0 or

b. an average MET gene copy number per cell of ≥6.0 or

c. ≥10% of tumor cells containing ≥15 MET signals.

2. *Intermediate-level* of gene copy number gain being defined as

a. ≥50% of cells containing ≥5 MET signals and

b. criteria for high-level amplification are not fulfilled

3. *Low-level* of gene copy number gain was defined as

a. ≥40% of tumor cells showing ≥4 MET signals and

b. criteria for high-level amplification or intermediate-level

of gene copy number gain are not fulfilled

4. All other tumors were classified as *negative*.

**Supplementary Tables**

**
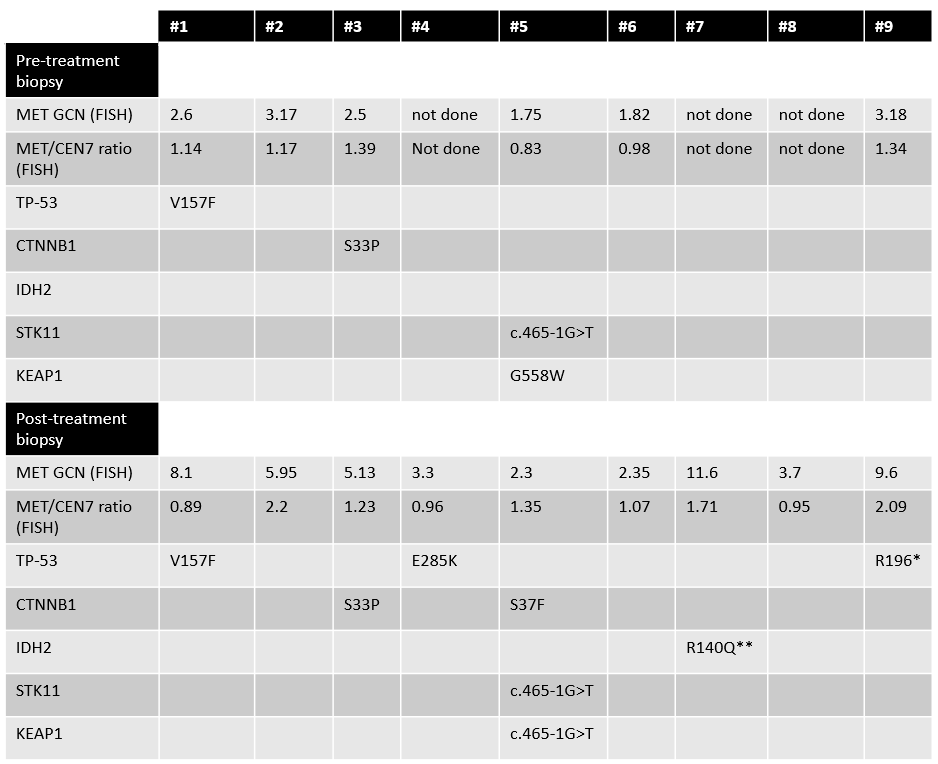
**

**Supplementary Table S1:** Summary of the results of molecular analyses of all nine patients.

GCN = gene copy number; MET/CEN7 = *MET* to centromer 7 ratio

|  | **Gene panels** | | | | | |
| --- | --- | --- | --- | --- | --- | --- |
| **Included genes** |  | LUN3 | LUN4 | LUN5 | nNGM 2.0 | nNGM 3.2 |
|  | AKT1 |  | not included | not included | not included | not included |
|  | ALK |  | not included |  |  |  |
|  | ARAF | not included |  | not included | not included | not included |
|  | BRAF |  |  |  |  |  |
|  | CTNNB1 |  |  |  |  |  |
|  | CUL3 | not included | not included | not included | not included |  |
|  | DDR2 |  |  | not included | not included | not included |
|  | EGFR |  |  |  |  |  |
|  | ERBB2 |  |  |  |  |  |
|  | FGFR1 | not included | not included |  |  |  |
|  | FGFR2 | not included |  |  |  |  |
|  | FGFR3 | not included |  |  |  |  |
|  | FGFR4 | not included | not included |  |  |  |
|  | HRAS | not included | not included | not included |  |  |
|  | IDH1 |  |  |  |  |  |
|  | IDH2 | not included | not included |  |  |  |
|  | KRAS |  |  |  |  |  |
|  | KEAP11 | not included |  | not included |  |  |
|  | MAP2K1 |  |  |  |  |  |
|  | MET |  |  |  |  |  |
|  | NFE2L2 | not included |  | not included | not included |  |
|  | NRAS | not included | not included |  |  |  |
|  | NTRK1 | not included | not included | not included |  |  |
|  | NTRK2 | not included | not included | not included |  |  |
|  | NTRK3 | not included | not included | not included |  |  |
|  | PIK3CA |  | not included | not included |  |  |
|  | PTEN |  |  | not included |  |  |
|  | ROS | not included | not included |  |  |  |
|  | RET | not included | not included | not included |  |  |
|  | STK11 | not included | not included | not included |  |  |
|  | TP53 |  |  |  |  |  |

**Supplementary Table S2:** NGS gene panels used.

**References**

1. Eisenhauer EA, Therasse P, Bogaerts J, Schwartz LH, Sargent D, Ford R, et al. New response evaluation criteria in solid tumours: revised RECIST guideline (version 1.1). Eur J Cancer. 2009;45(2):228–47.
2. Wahl RL, Jacene H, Kasamon Y, Lodge MA. From RECIST to PERCIST: evolving considerations for PET response criteria in solid tumors. J Nucl Med. 2009;50 Suppl 1:122S–150S.
3. Michels S, Heydt C, Brüning T, Scheel AH, Büttner R, Merkelbach-Bruse S, et al. Genomic profiling identifies outcome-relevant mechanisms of innate and acquired resistance to third-generation epidermal growth factor receptor tyrosine kinase inhibitor therapy in lung cancer. JCO Precis Oncol. 2019;3:1–15.
4. Peifer M, Fernández-Cuesta L, Sos ML, George J, Seidel D, Kasper LH, et al. Integrative genome analyses identify key somatic driver mutations of small-cell lung cancer. Nat Genet. 2012;44(10):1104–10.
5. Schildhaus HU, Schultheis AM, Rüschoff J, Binot E, Merkelbach-Bruse S, Fassunke J, et al. MET amplification status in therapy-naïve adeno- and squamous cell carcinomas of the lung. Clin Cancer Res. 2015;21(4):907–15.
